# Dopamine receptor genetic polymorphisms and body composition in undernourished pastoralists: An exploration of nutrition indices among nomadic and recently settled Ariaal men of northern Kenya

**DOI:** 10.1186/1471-2148-8-173

**Published:** 2008-06-10

**Authors:** Dan TA Eisenberg, Benjamin Campbell, Peter B Gray, Michael D Sorenson

**Affiliations:** 1 Department of Anthropology, Northwestern University, Evanston, IL, USA; 2 Department of Anthropology, University of Wisconsin Milwaukee, Milwaukee, WI, USA; 3 Department of Anthropology and Ethnic Studies, University of Nevada, Las Vegas, USA; 4 Department of Biology, Boston University, Boston, MA, USA

## Abstract

**Background:**

Minor alleles of the human dopamine receptor polymorphisms, DRD2/TaqI A and DRD4/48 bp, are related to decreased functioning and/or numbers of their respective receptors and have been shown to be correlated with body mass, height and food craving. In addition, the 7R minor allele of the DRD4 gene is at a higher frequency in nomadic compared to sedentary populations. Here we examine polymorphisms in the DRD2 and DRD4 genes with respect to body mass index (BMI) and height among men in two populations of Ariaal pastoralists, one recently settled (n = 87) and the other still nomadic (n = 65). The Ariaal live in northern Kenya, are chronically undernourished and are divided socially among age-sets.

**Results:**

Frequencies of the DRD4/7R and DRD2/A1 alleles were 19.4% and 28.2%, respectively and did not differ between the nomadic and settled populations. BMI was higher in those with one or two DRD4/7R alleles in the nomadic population, but lower among the settled. Post-hoc analysis suggests that the DRD4 differences in BMI were due primarily to differences in fat free body mass. Height was unrelated to either DRD2/TaqI A or DRD4/48 bp genotypes.

**Conclusion:**

Our results indicate that the DRD4/7R allele may be more advantageous among nomadic than settled Ariaal men. This result suggests that a selective advantage mediated through behaviour may be responsible for the higher frequency of the 7R alleles in nomadic relative to sedentary populations around the world. In contrast to previous work, we did not find an association between DRD2 genotypes and height. Our results support the idea that human phenotypic expression of genotypes should be rigorously evaluated in diverse environments and genetic backgrounds.

## Background

Genetic variations in the dopamine (DA) system have been related to nutritional indices [1-11] and a nomadic lifestyle [12]. While human neurological, behavioural and physiological genetics is a vast field, little such research has been conducted among people living in non-industrialized or subsistence environments. Such environments may be more similar to the environments where much of human genetic evolution took place; indeed, they may be adaptively relevant environments [AREs; [13]] for the evolution of dopamine gene polymorphisms that have been primarily investigated in industrialized settings. Here we examine the relationships between genetic polymorphisms of dopamine receptor genes and several phenotypes in Ariaal men.

The Ariaal are traditionally nomadic pastoralists living in northern Kenya. They are mainly a subsistence population with low percent body-fat and chronic under-nutrition [14]. Roughly half of our sample consists of nomads, while the other half are from a group that has been settled for about 35 years and practices some agriculture [15,16]. This contrast between nomadic and settled Ariaal groups provides an opportunity to test for gene by environment interactions across two environments inhabited by genetically and culturally similar peoples. We examine how two dopamine receptor genetic polymorphisms relate to measures of nutrition/body composition among these two groups. To the best of our knowledge, this is the first study to examine correlates of dopamine genetic variation in a subsistence society.

### Dopamine gene polymorphisms and their correlates

This study analyzes the correlates of two genetic polymorphisms, the TaqI A polymorphism in the dopamine receptor D2 (DRD2) gene and the 48 base pair (bp) repeat polymorphism in the dopamine receptor D4 (DRD4) gene. There is evidence that minor alleles of both DRD2 and DRD4 (A1 and 7R respectively) decrease the sensitivity and/or concentrations of their respective receptors [17-24]. Thus, minor alleles can be viewed as analogous to their respective dopamine receptor antagonists (although this neglects developmental effects), and thus provide natural experiments to dissect aspects of human physiology. DRD2 and DRD4 are both considered D2-like receptors and have similar functions and distributions, but are distinct. DRD2 seems to be particularly important in the striatum, whereas DRD4 appears more important in the prefrontal cortex [reviewed in [25]]. Both are likely involved in impulsivity, reward anticipation and addiction and they may interact in a complex manner to effect phenotypes [reviewed in [25,26]].

The DRD4/48 bp polymorphism has been associated with body mass index [BMI = weight in kg/height in meters^2; [9-11], however [27]] and food craving [28]. The allele frequencies of DRD4/48 bp vary considerable across populations and the 7R minor allele is generally at a higher frequency in populations that have migrated farther or are nomadic rather than sedentary [12] and among individuals with multi-racial ancestries [29]. It appears that the 7R allele emerged and began being positively selected for about 45,000 years ago [30].

The A1 allele of the DRD2/TaqI A polymorphism has often been associated with substance abuse [18]. The DRD2 gene has also been variously related to BMI and related indices of metabolic syndrome [1-8,31]. Lower striatal D2 receptor availability has been related to obesity and increased BMI [32]. Those with DRD2 A1 alleles have higher food reinforcement (are willing to work harder for food) and consume more food than their counterparts without A1 alleles [33,34], suggesting DRD2 impacts nutritional status through food craving behaviour.

Increases in height have been reported among children who were exposed pre- or post-natally to D2 receptor blocking drugs [reviewed in [35]]. Additionally, among four independent sets of subjects DRD2 polymorphisms were related to height [35-37].

### Predictions and findings

Based on past research suggesting that BMI associations with dopamine receptor polymorphisms are due to increased food craving [36,38], we expected that BMI would be related to dopamine receptor genotype. However, food craving in western environments of plenty might have very different implications than among undernourished Ariaal men. Based on observations that nomads around the world have higher DRD4/7R allele frequencies relative to settled populations [12], we predicted that 7R+ nomads would have better relative indices of nutrition than 7R+ settled Ariaal.

We compared allele frequencies between the settled and nomadic men, but due to the settled group's recent formation [15] and continued gene flow with the nomads, we did not have clear expectations for allele frequency differences.

We also expected that DRD2 would be associated with height as shown in previous research. Since height is a key variable in calculating BMI and DRD2 and DRD4 are functionally related, height was examined in relation to both DRD2 and DRD4.

We found that BMI was higher in individuals with one or two 7R alleles in the nomadic population, but was lower in settled individuals with the 7R allele. Post-hoc analysis suggests that these DRD4-related differences in BMI were mainly due to differences in fat free mass. Height was unrelated to dopamine genetic polymorphisms.

## Results and Discussion

### Allele and genotype frequencies in nomadic and settled men

DRD4 allele and genotype frequencies are shown in Table 1. Settled and nomadic Ariaal did not differ in their 7R allele frequencies (Pearson chi-square = 0.26, df = 1, p = 0.610) nor were there significant differences in overall allele frequencies (Fisher's exact test, p = 0.906). The DRD4 genotype frequencies of the settled and nomadic Ariaal pooled together were in Hardy-Weinberg equilibrium (HWE; Markov Chain algorithm, p = 0.124) as well as in the nomads alone (p = 0.735). The DRD4 genotype frequency among settled Ariaal was slightly out of HWE (Markov Chain algorithm, p = 0.036), but when the single 5R/5R individual was excluded from the analysis, HWE was maintained (p = 0.425). No 2R alleles, the third most common and likely functionally distinct allele, were found in either the nomadic or sedentary samples; consistent with a lack of 2R alleles in some other African populations [39]. DRD2 allele and genotype frequencies are shown in Table 2. The DRD2 genotype frequencies of the settled and nomadic Ariaal pooled together was in HWE (Fisher's exact test, p = 0.554) as well as for the settled (Fisher's Exact, p = 0.135) and nomadic groups separately (Fisher's Exact, p = .526).

**Table 1:** Allele and genotype frequencies for DRD4/48 bp polymorphism.

	All		Settled		Nomadic	
**Allele/Genotype**	**n**	**%**	**n**	**%**	**n**	**%**

Allele						
4	232	76.3	130	74.7	102	78.5
5	3	1.0	2	1.1	1	0.8
6	4	1.3	2	1.1	2	1.5
7	59	19.4	36	20.7	23	17.7
8	2	0.7	1	0.6	1	0.8
10	4	1.3	3	1.7	1	0.8
Total	304	100.0	174	100.0	130	100.0
Genotype Classification						
4/4	89	58.6	49	56.3	40	61.5
4/5	1	0.7	0	0.0	1	1.5
4/6	4	2.6	2	2.3	2	3.1
4/7	45	29.6	28	32.2	17	26.2
4/8	1	0.7	0	0.0	1	1.5
4/10	3	2.0	2	2.3	1	1.5
5/5	1	0.7	1	1.1	0	0.0
7/7	6	3.9	3	3.4	3	4.6
7/8	1	0.7	1	1.1	0	0.0
7/10	1	0.7	1	1.1	0	0.0
7-	99	65.1	54	62.1	45	69.2
7+	53	34.9	33	37.9	20	30.8
Total	152.0	100.0	87.0	100.0	65.0	100.0

**Table 2:** Allele and genotype frequencies for the DRD2/TaqI A polymorphism.

	All		Settled		Nomadic	
**Allele/Genotype**	**n**	**%**	**n**	**%**	**n**	**%**

Allele						
A1	88	28.2	53	30.5	35	25.4
A2	224	71.8	121	69.5	103	74.6
Total	312	100	174	100	138	100
Genotype Classification						
A1/A1	14	9.0	11	12.6	3	4.3
A1/A2	60	38.5	31	35.6	29	42.0
A2/A2	82	52.6	45	51.7	37	53.6
A1+	74	47.4	42	48.3	32	46.4
A1-	82	52.6	45	51.7	37	53.6
Total	156.0	100.0	87.0	100.0	69.0	100.0

### Body Composition/Nutritional Status

**Figure 1:**
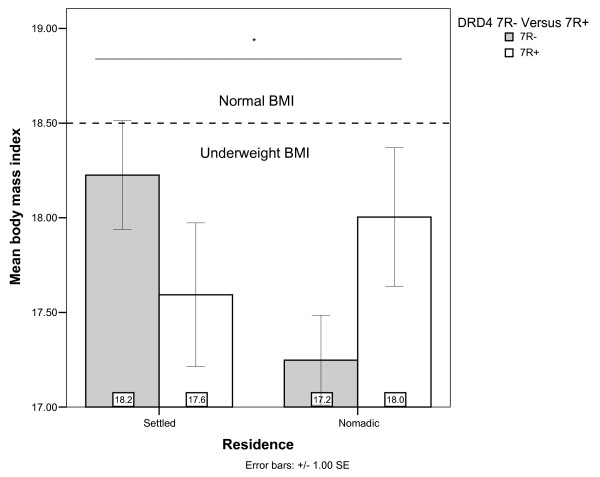
**Body Mass Index by residence and DRD4 genotype**. *Residence × DRD4 interaction significant (Table 4).

Descriptions of the anthropometric variables used in this study, including BMI and height, are given in Table 3 for the pooled population and two sub-populations. The nomadic and settled groups of men that make up the sample are on average underweight, having BMIs below the 18.5 standard of normal-weight [40,41]. General Linear Models to evaluate the relation of DRD2 and DRD4 with anthropometrics first included DRD2, DRD4, residence and all two-way interactions and then, in a separate model, included variables shown in past studies to be associated with the anthropometric measure of interest [14,42,43]. As can be seen in Models 1 & 2 of Table 4, a strong interaction between DRD4 and residence with BMI was evident. The nature of this interaction between DRD4 and residence is illustrated in Figure 1, where among nomads 7R+ was related to being less underweight (higher BMI), but among settled men, 7R+ individuals were more underweight (lower BMI).

**Table 3:** Descriptions of phenotype variables. Abbreviations are defined in list of abbreviations.

	All		Settled		Nomadic	
**Phenotype**	**mean**	**SD**	**Mean**	**SD**	**Mean**	**SD**

n	152		87		65	
Age	46.85	16.49	47.22	16.46	46.35	16.65
BMI	17.77	1.95	17.99	2.15	17.48	1.63
Height (cm)	172.32	6.63	172.20	6.69	172.47	6.60
AMPBA	37.46	6.26	37.49	6.10	37.42	6.50
TSK	4.85	1.79	4.95	2.04	4.73	1.39
% BF	9.62	3.12	9.67	3.26	9.54	2.97
FFM (kg)	47.45	5.49	47.76	5.58	47.04	5.39
SSK	4.72	2.11	5.11	2.65	4.19	0.75

**Table 4:** Correlates of Body Composition (General Linear Models). Abbreviations are defined in list of abbreviations. Significant and near significant associations are bolded.

Model	1	2 Φ	3	4	5	6	7	8	9	10	11 Φ	12	13
**Predictor**	**BMI**	**BMI**	**Height**	**Height**	**AMPBA**	**AMPBA**	**TSK**	**%BF**	**%BF**	**FFM**	**FFM**	**SSK**	**SSK**

*Adj. r*^2^	0.03	0.10	-0.02	0.03	0.02	0.01	0.01	-0.02	0.11	0.03	0.20	0.05	0.14
Residence	0.64	0.03	0.38	0.05	0.21	0.03	0.32	0.06	0.06	0.03	0.02	**7.61	4.49
DRD2	1.62	0.62	0.33	0.81	1.64	1.06	0.80	0.40	0.77	2.53	0.69	0.27	0.26
DRD4	0.09	0.12	0.73	0.84	2.22	1.13	0.46	0.32	0.07	2.06	2.09	0.55	0.78
DRD2 × DRD4	0.63	0.46	0.00	0.18	0.38	1.05	2.08	1.66	0.83	0.01	0.09	**†3.42**	2.19
DRD2 × Residence	0.48	0.98	0.25	0.02	**†2.801**	1.65	0.37	0.01	0.00	0.49	1.47	0.68	0.30
DRD4 × Residence	*5.21	**7.64	1.99	2.59	*3.921	**†2.94**	1.45	0.61	0.71	**7.17	***11.64	0.07	0.34
Age Group	-	1.10	-	-	-	-	-	-	***6.49	-	***5.72	-	-
PM T	-	**9.42	-	1.36	-	0.75	-	-	0.34	-	*5.38	-	***15.65
AR	-	1.21	-	**9.53	-	-	-	-	0.20	-	*6.21	-	*4.62
PM T × AR	-	**†3.51**	-	**7.42	-	-	-	-	0.10	-	**7.12	-	**7.16

†p ≤ 0.1; *p ≤ 0.05; **p ≤ 0.01; ***p ≤ 0.001

Φ Detailed statistics for models 2 and 11 are given in additional files 3 and 4 respectively.

Height did not show the predicted association with DRD2, nor was height associated with DRD4 (Table 4; Models 3 and 4). Closer examination of the only other study specifically testing for an association between DRD2/TaqI A and height suggests that the A1 allele may differ in its linkages in different populations. In that study, idiopathic short stature was associated with A1 alleles in Japanese subjects [37]. *Increases *in height have been reported among children who were exposed pre or post-natally to D2 receptor blocking drugs [reviewed in [35]]. However, the A1 allele of the DRD2/TaqIA polymorphism is generally associated with *decreased *DRD2 functioning/expression [reviewed in [25]], so together with the D2 receptor blocking studies, we should expect A1 alleles to be associated with *increased *(not decreased) height. Since the A1 allele is not thought to be a functional polymorphism in the dopamine system (elaborated in the methods section), varying genetic background (i.e. different patterns of linkage with actual functional genetic polymorphisms) could explain varying results across populations.

Since BMI is a coarse measure of body composition we conducted further post-hoc analysis in an effort to understand the associations of DRD4 with body composition at a finer level. We analyzed upper arm muscle area plus bone area (AMPBA) because it represents short term energy balance/storage [44] and low values of AMPBA in the elderly are associated with increased mortality [45], triceps skinfold (TSK) as a measure of energy stores and current energy balance [44], percent body fat (%BF) as a measure of general adiposity, fat free mass (FFM) as another measure of body size, and suprailliac skinfold (SSK) as a measure of abdominal fat, thought to be the most metabolically active fat depot [46].

**Figure 2:**
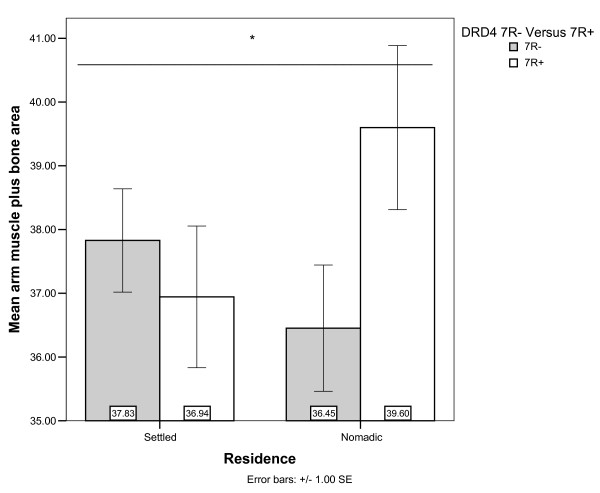
**Arm Muscle Plus Bone Area by residence and DRD4 genotype**. *Residence × DRD4 interaction significant in model 5, but not model 6 (Table 4).

**Figure 3:**
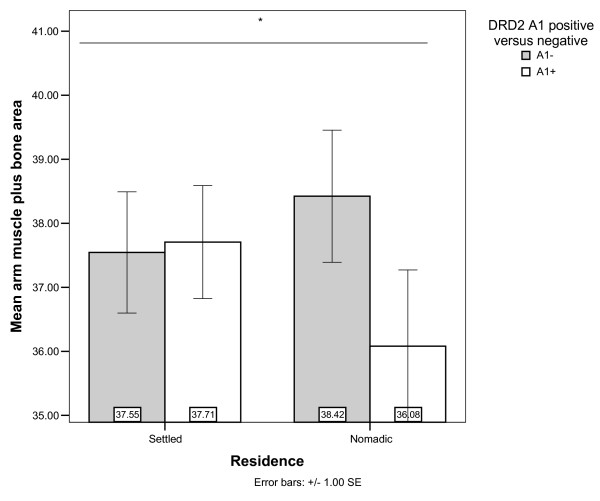
**Arm Muscle Plus Bone Area by residence and DRD2 genotype**. *Residence × DRD2 interaction near significant in Model 5 and non-significant in Model 6 (Table 4).

AMPBA might be associated with DRD4 and DRD2 through interactions with residence, although the effect significance depends on the other covariates in the models (Table 4, Models 5 and 6). Nomads with 7R alleles had higher AMPBA, whereas little or no difference was found in settled individuals (Figure 2). Based on an expectation of similar effects of DRD2 and DRD4 minor alleles, it is peculiar that the DRD2 A1+ genotype was near significantly associated with decreased AMPBA among the nomads (Figure 3). Perhaps this can be understood as effects of DRD2 on GH and IGF-1 mediated muscle formation [37,47] or maintenance [48,49]. However, such an explanation of DRD2's effect on AMPBA is not consistent with our suggested explanation for the lack of association of DRD2 with height.

**Figure 4:**
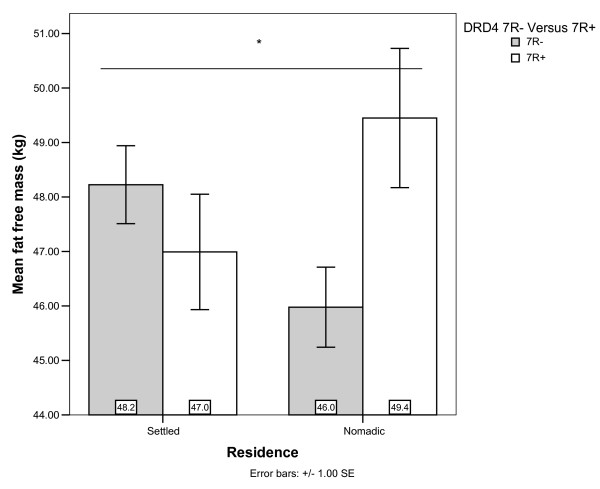
**Fat Free Mass (in kg) by residence and DRD4 genotype**. *Residence × DRD4 interaction significant (Models 10 & 11; Table 4).

In models 7–9, 12 & 13 (Table 4), we show that TSK, %BF and SSK were not related to DRD2 or DRD4 genotypes. However, models 10 & 11 (Table 4), show a relatively strong association between the interaction of DRD4 and residence with fat free mass (FFM). Inclusion of additional relevant covariates increases the significance of this interaction and the explanatory power of the model (adjusted r^2^). The direction of the association with FFM (Figure 4) was the same as with BMI (Figure 1); nomads with 7R alleles had higher FFM than their nomadic counterparts without 7R alleles, and the effect of DRD4 was reversed and of a lesser magnitude among the settled. Since FFM has previously been shown to vary with age group [Table 4, Model 11; [42]], we additionally checked for DRD4 by age group and DRD4 by residence by age group interactions, but these were not evident (not shown).

Table 5 is provided to explore the inheritance patterns (e.g. recessive, dominant, additive or other) of the associations of DRD4 with BMI and FFM. The association of DRD4 with BMI seems to exhibit an additive relationship; as the number of 7R alleles increases from zero 7R alleles (7R-/7R-), to one 7R allele (7R/7R-), to two (7R/7R), BMI decreases in the settled men, but increases in the nomads. FFM, which was more closely related to DRD4, does not exhibit as clear a trend. This analysis is obviously limited by the low sample sizes, especially among 7R homozygotes.

**Table 5:** BMI and FFM by three DRD4 genotypes and settlement patterns.

		Settled		Nomadic	
Genotype	Measure	BMI	FFM	BMI	FFM

7R-/7R-	Mean	18.23	48.22	17.25	45.98
	n	54	54	45	45
	SD	2.11	5.25	1.59	4.93
7R/7R-	Mean	17.67	47.05	17.99	49.50
	n	30	30	17	17
	SD	2.26	6.36	1.75	5.93
7R/7R	Mean	16.78	46.36	18.06	49.13
	n	3	3	3	3
	SD	0.96	2.26	0.97	5.43

DRD4 7R+ seems to increase total non-fat mass (FFM) of the body, but only among nomads. FFM is defined as bodyweight minus body-fat weight and thus primarily measures the sum of the masses of muscle, bone, and organs. With this exclusion of fat mass, FFM represents the more metabolically active body portions [50]. FFM is a general index of body-size and in a chronically underweight population, can reasonably be assumed to represent greater body-size and/or greater muscular and bone mass. However, given the lack of association of DRD4 with height, the latter suggestion of increased muscle and bone mass seems more likely. In an undernourished population with low % body fat (Table 3), increased muscle and bone mass might actually represent less starvation induced breakdown of muscle mass and higher nutrient stores (perhaps due to nutritional protein deficiency) [51].

The finding that the 7R allele had a positive association with BMI, FFM and AMPBA among nomads, but a negative one among settled individuals is consistent with past findings of higher 7R allele frequencies in nomadic populations around the world [12]. While dopaminergic pathways clearly are involved in food craving (see above), the significance of increased food cravings is unclear in a nutrient poor environment, where food to satisfy such cravings is not readily available. That 7R alleles are associated with selfishness [52] might also mean that while craving food more those 7R men are also less likely to share food. However, it is unclear why food craving or selfishness should vary in its outcome by residence among the Ariaal.

HIV infection, common in Kenya, can cause wasting. Past study among the Ariaal suggest low rates of condom usage and a cultural acceptability for men to have multiple simultaneous sexual partners [53]. Sexual behaviours have been related to DRD4 [29,54,55] which might alter sexually-transmitted disease risks. However, information on HIV infection rates among the Ariaal is not available.

Increased impulsivity, ADHD-like traits, novelty-seeking like traits [reviewed in [25]], aggression [56,57], violence [26] and/or activity levels [58,59] may help nomads obtain food resources, or exhibit a degree of behavioural unpredictability [60] that is protective against interpersonal violence or robberies [61,62]. Increased activity levels, if not resulting in increased food access, likely would decrease nutritional stores. The 7R allele might also promote better infant survival [63-66] depending upon the social and ecological context. Alternatively, these associations may represent some other more basic metabolic influence of DRD4, such as regulation of physical activity in high temperature environments [67-69]. Regardless of the mechanism, the fact that the DRD4 7R allele remains at relatively low frequency (less than 20%) despite indications that it contributes to improved somatic condition among nomads, suggests that the 4R/7R polymorphism is maintained by frequency dependent selection [62,70] (or perhaps, but less likely heterozygote advantage [71]).

We recognize that both DRD2 and DRD4 genotypes are sometimes parsed differently than shown here. To allow further analysis and comparisons with other studies, we have included the complete dataset used for this manuscript as Additional files 1 &2. As well, full statistics, effect sizes and observed power for models 2 and 11 from Table 4 are given in Additional files 3 and 4 respectively.

## Conclusion

The DRD2 gene was not associated with height in this non-industrialized group – at variance with four previous studies. We note that the TaqI A polymorphism is itself not know to be functional (see methods), so this finding could be due to differences in genetic linkage by population. Re-sequencing or more extensive haplotype analysis of this region in this and other populations might elucidate the matter. Alternatively, population specific epistatic interactions or the chronic undernutrition experienced by the Ariaal men in this sample might alter the relative expression/importance of DRD2 genotypes in determining height.

We found that DRD4 7R+ genotypes were associated with indices of better nutritional status among nomads, particularly higher fat free mass, but worse indices in the settled individuals. This suggests that the 7R allele confers additional adaptive benefits in the nomadic compared to sedentary context. These benefits might be derived from behavioural or metabolic differences between genotypes that vary either in their physiological/psychological phenotypic expression or practical result by context. Future study will be required to distinguish between potential mechanisms involved.

In order to elucidate the current finding, future studies among the Ariaal might look more specifically at food intake, attention in children, mechanisms of defence of livestock and/or activity levels. It might be that the attention spans conferred by the DRD4/7R+ genotype allow nomadic children to more readily learn effectively in a dynamic environment (without schools), while the same attention span interferes with classroom learning in Songa, the settled community. 7R+ boys might develop into warriors (the life-stage of an Ariaal male that lies between childhood and manhood) and men who can more effectively defend against livestock raiders, perhaps through a reputation of unpredictable behaviour that inspires fear. Among 7R+ men in the settled community of Songa, such tendencies might be less well suited to practicing agriculture and selling goods at market. It might also be that higher activity levels in 7R+ nomads are translated into increased food production, while such activity levels in settled men are a less efficient use of calories in food production.

This study highlights the importance of studying human biology, and genetic polymorphisms in particular, in diverse environments and among genetically distinct populations.

## Methods

### Populations and Field Work

The Ariaal are pastoralists inhabiting both upland and lowland regions in the Marsabit district of Kenya. First appearing in oral history in the 1880s, they are derived from a group of poor Rendille and Samburu groups who banded together to build up their herds in the mountains. Culturally, they still exhibit features of both Rendille and Samburu, including Samburu age-set rituals and Rendille annual camel blessings [15]. Ariaal derive their subsistence from herding camels, cattle, goats, and sheep, which they depend on for nutrition in the form of milk, blood, and meat.

In August of 2005, as part of a study on male aging, Ariaal participants were recruited from two venues: 103 men from the settled agropastoral community of Songa and 102 nomadic males from the nomadic settlement of Lewogoso on the Kasuit Plateau. Potential subjects were identified by a scattershot method (no selection criteria) in the settled and nomadic community, and the final sample stratified by ten year age groups; 20–29, 30–39, 40–49, 50–59, 60+.

In Songa, maize and other foodstuffs make important contributions to the diet [15]. The existence of drip-irrigation supports the production of crops including oranges that are sold in the nearby town of Marsabit, approximately 45 minutes away. Songa also includes an elementary school and a nurse's station. In the nomadic community, Lewogoso, all eligible men were asked to participate. Lewogoso is located approximately 45 minutes from the Settlement of Korr and 3 hours from the town of Marsabit by vehicle. The nearest clinic is in Ngrunit about 40 minutes away. Children do not attend elementary school.

Interviews, assisted by trained translators in the appropriate language (Samburu and/or Rendille), were conducted with each participant. Interviews focused on demographic background, marital and reproductive history, social support and age-related quality of life outcomes. During interviews, ages were estimated with reference to an event calendar and age set membership, and further ambiguities checked with local assistants [for further details see [72]].

### Anthropometrics, genetic and hormone measures

#### Anthropometrics

Anthropometric measures included height, weight, arm, waist and hip circumference and five skinfolds: triceps, subscapular, midaxillary, suprailliac and periumbilical. All anthropometric measures were taken by an experienced human biologist (BC). Derived measures include % body fat, calculated from skinfolds based on the D-W equations [73]. Because the D-W equations are based on a Caucasian sample, they may provide absolute values of % body fat that are not directly comparable with other populations. However, they should provide a relatively consistent measure of % body fat within the study population considered here. We also derived arm muscle plus bone area (AMPBA), calculated as ([MUAC - (π × TCSF/10)]^2^)/4 where MUAC is mid upper arm circumference in cm and TCSF is the triceps skinfold in mm [74]. Fat free mass (FFM) was calculated as weight * ([100 - % body fat]/100).

#### Genotyping

Hair samples with roots were obtained by plucking, and immediately placed in zip lock bags for transportation to the lab of MDS. We obtained hair samples from 156 men, including 87 settled and 69 nomadic men. The other men lacked sufficient hair to obtain a sample, either because of baldness or the fact that they had shaved their heads. Samples were stored at ambient temperature in the field and then in a -20°C freezer until analysis. Bulbs were removed from hairs under a dissecting microscope, and DNA was extracted with a DNeasy Tissue Kit (Qiagen) and eluted in 200 μl of tris elution buffer.

#### DRD4 48 bp VNTR

The *DRD4 48-bp VNTR *polymorphism is in exon 3 of the gene coding for the dopamine D4 receptor. The *VNTR *polymorphism varies between 2 and 11 repeats of a similar *48 bp *coding region sequence, with a tri-modal distribution of 2, 4 and 7 repeat alleles (2R, 4R and 7R) in most, but not all, populations [75]. While the functional significance of the *DRD4 VNTR *polymorphism has not been definitively characterized, long alleles (typically 7R as opposed to 4R) might be functionally less reactive based on in-vitro expression experiments [20-24], however much heterogeneity still exists [76-80]. Similarly, in vivo human pharmacological studies are generally consistent with the notion that 7R alleles are associated with less responsive D4 receptors than 4R alleles [81-85].

The *DRD4 48 bp VNTR *locus was genotyped using an adaptation of a previous protocol [25,86]. The PCR reaction contained 1× Q-Solution (Qiagen), 1× PCR Buffer (Qiagen), 1 μM Primer F1 (5' GCGACTACGTGGTCTACTCG 3'), 1 μM Primer R1 (5' AGGACCCTCATGGCCTTG 3'), 200 μM dATP, dTTP, dCTP and 100 μM dITP and dGTP, 0.3 units HotStar Taq (Qiagen), 1 to 5 μl of DNA template, in a total volume of 10 μl. Thermocycler conditions were: 15 min at 95° to activate the enzyme and denature the DNA, 40 cycles of 1 minute denaturation at 94°, 1 minute annealing at 55°, 1.5 minute extension at 72°, followed by one cycle of 10 minute extension at 72°. With these primers, a 4R PCR product is 475 bp. Samples were visualized under UV on 2.0% ethidium bromide agarose gels with a 100 bp ladder. In contrast to previous results using this protocol [25], varying concentrations of the DNA template did not result in allelic dropout. However, as observed previously at this locus [87] extra heteroduplex bands formed in several samples. To confirm that these bands were in fact PCR artefacts, two sets of alternative primers were used under identical conditions (F2 5' CGTACTGTGCGGCCTCAACGA 3' and R2 5' GACACAGCGCCTGCGTGATGT 3', 705 bp product for 4R [75]; and F3 5' CTGCAGCGCTGGGAGGTG 3' and the R1 primer above, 389 bp product for 4R).

#### DRD2 TaqI A

The *DRD2 TaqI A *site is a single-nucleotide polymorphism with a major A2, and minor A1 allele. The A1+ genotype (heterozygous or homozygous for A1) has been most strongly associated with substance abuse, particularly alcoholism, albeit with some ambiguity [18]. Although the mechanisms remain poorly understood, the A1 allele is associated with decreases in dopamine D2 binding and glucose metabolic rates in many brain regions [17-19]. The *DRD2 TaqI A *locus is 9.4 kb downstream from the coding region for the *DRD2 *gene, is not in any known regulatory region, and has no known mechanism to influence *DRD2 *expression. The *TaqI A *polymorphism is also in a nearby kinase gene, the *Ankyrin Repeat and Kinase Domain Containing 1 (ANKK1) *gene, where it causes a Glutamate→Lysine substitution [88,89]. The results of the amino acid substitution are not known, but could impact interactions of ANKK1 proteins with other proteins including the dopamine D2 receptor [89]. No other polymorphism has been revealed to be in linkage disequilibrium with *TaqI *A that could clearly account for its functional associations [88,89].

*DRD2 TaqI A *was typed with a PCR/RFLP method [based on [25,90]]. The PCR reaction contained 0.5 μM forward (5' CACGGCTGGCCAAGTTGTCTA 3'), 0.5 μM reverse primers (5' CACCTTCCTGAGTGTCATCAA3'), 1.25 mM dNTP, 2.5 mM MgCl_2_, 0.5 units AmpliTaq Gold (ABI), 1× Buffer (ABI), 2 μl DNA template in a total volume of 20 μl. Cycle conditions were: 8 min denaturation at 95°C, 40 cycles of 30 s denaturation at 94°, 30 s annealing at 55°C, 1.5 min extension at 72°C and one final extension of 7 minutes at 72°. The PCR product was digested with *Taq*I enzyme (New England Biolabs) for at least two hours at 65°C and visualized under UV on a 1% ethidium bromide agarose gel. The 300 bp PCR product is not cut by the restriction enzyme in A1 homozygotes, whereas the A2 allele yields 125 and 175 bp fragments. PCR products for a sample of A1 homozygotes were re-digested to assure proper genotyping.

#### Androgen Receptor (AR) CAG tri-nucleotide repeat

The AR CAG microsatellite was typed with a PCR mix containing 1.25 μM forward primer (TCCAGAATCTGTTCCAGAGCGTGC) and 1.25 μM reverse primer (GCTGTGAAGGTTGCTGTTCCTCAT), 2.5 mM dNTP, 2.5 mM MgCl_2_, 0.625 units AmpliTaq Gold (ABI), 1× Buffer (ABI), 3 μl DNA template in a total volume of 25 μl. The reverse AR primer was fluorescently labelled with GeneScan VIC (ABI). Cycle conditions were: 8 min denaturation at 95°C, 40 cycles of 30 s denaturation at 95°, 30 s annealing at 55°, 1 min extension at 72° and one final extension of 7 minutes at 72°. PCR products were analyzed on an ABI 3100 genetic analyzer using GeneMapper 3.7 (ABI). Four random samples were sequenced for length confirmation. The number of trinucleotide repeats was calculated by subtracting 214 bp from the product then dividing by three. The number of repeats ranged from 15–34. A median split at 20 (<= 20 vs. >20) was used for analyses as in [43].

#### Salivary Testosterone

Saliva samples were collected using standard methods [91] including stimulation of saliva with Original flavoured Carefree gum. Morning samples were collected within 15 minutes of 09:00 hours, while afternoon saliva samples were collected within 15 minutes of 16:00 hours. Sodium azide was added as a preservative and samples were transported to the Laboratory of Reproductive Ecology at Harvard University where they were assayed using previously described RIA techniques [42]. The interassay coefficient of variation was 15%. Four morning T values and two afternoon T values were more than three SDs from their respective means, and were eliminated from the analyses. Analyses here only use afternoon T values to be consistent with past studies, however results were essentially unchanged when morning T values were included in the models instead (not shown).

### Statistical Analysis

All anthropometric variables were examined for distribution normality. HW equilibriums were tested with the HWE program [92]. All other statistical analyses were conducted with SPSS 11. *DRD2 *HW equilibrium was tested with Fisher's exact test and *DRD4 *was tested with the Markov Chain algorithm [93]. Two-tailed p values less than or equal to 0.05 were considered significant and values less than or equal to 0.10 were considered near significant.

Based on previous association studies, for the *DRD2 TaqI A*, individuals with at least one A1 allele were designated as A1+ and those who were homozygous for the A2 allele were designated A1-. Similarly, DRD4 VNTR genotypes were separated into 7R allele present (7+) and 7R absent (7-) groups. Since we recognize that other methods of parsing genotype groups and analyzing the data may be legitimate and further our understanding, we have included, as Additional file 1 and 2, the complete dataset on which this manuscript is based. The analyses used 2 (Settled/Nomadic) × 2 (A1+/A1-) × 2 (7+/7-) General Linear Models (using GLM function in SPSS; including all primary and 2-way effects).

When past results indicated the outcome variable of interest was related to other predictor variables, another model was built which also included the appropriate additional variables. These other variables consisted of testosterone level (afternoon measurement), the androgen receptor (AR) genetic polymorphism, and age group.

## List of abbreviations

%BF: Percent Body Fat; AMPBA: Arm Muscle Plus Bone Area; AR: Androgen Receptor genetic polymorphism-CAG trinucleotide repeat; bp: base pair; BMI: Body Mass Index (weight in kg/m^2^); DA: dopamine; DRD2: Dopamine Receptor D2; DRD4: Dopamine Receptor D4; FFM: Fat Free Mass; GH: growth hormone; HWE: Hardy-Weinberg Equilibrium; IGF-I: Insulin like Growth Factor I; Pm T: Testosterone levels taken in the afternoon; Residence: whether the subject is from a nomadic or sedentary community; SSK: Suprailliac Skinfold; TSK: Triceps Skinfold.

## Authors' contributions

DTAE co-conceived of the study, carried out genetic laboratory analysis, helped develop genetic laboratory protocols, analyzed the data and wrote the manuscript. BC co-conceived the study, collected samples and phenotype data and assisted in analysis of data and manuscript preparation. PBG collected samples and phenotype data. MDS supervised genetic laboratory analyses, designed primers and helped develop genetic laboratory protocols. All authors read and approved the final manuscript.

## Supplementary Material

Additional file 1 **Dataset**. Entire dataset used for analysis in this manuscript and some additional variables Click here for file

Additional file 2 **Dataset Legend**. Description of variables (column headings) given in Additional file 1. This file also contains descriptions of some variables NOT included in Additional file 1. Those interested in those variables not included in file 1 should contact the authors. Click here for file

Additional file 3 **Details of Model 2 from **Table 4**– BMI**. Full statistical model of model 2 from table 4. Dependent variable is BMI. Click here for file

Additional file 4 **Details of Model 11 from **Table 4**– FFM**. Full statistical model of model 11 from table 4. Dependent variable is FFM. Click here for file
